# Identification of Gene Expression Changes Associated With Uterine Receptivity in Mice

**DOI:** 10.3389/fphys.2019.00125

**Published:** 2019-02-14

**Authors:** Jia-Peng He, Miao Zhao, Wen-Qian Zhang, Ming-Yu Huang, Can Zhu, Hao-Zhuang Cheng, Ji-Long Liu

**Affiliations:** College of Veterinary Medicine, South China Agricultural University, Guangzhou, China

**Keywords:** uterus, receptivity, mouse, RNA-seq, gene expression

## Abstract

The mouse is a widely used animal model for studying human reproduction. Although global gene expression changes associated with human uterine receptivity have been determined by independent groups, the same studies in the mouse are scarce. The extent of similarities/differences between mice and humans on uterine receptivity at the molecular level remains to be determined. In the present study, we analyzed global gene expression changes in receptive uterus on day 4 of pregnancy compared to non-receptive uterus on day 3 of pregnancy in mice. A total of 541 differentially expressed genes were identified, of which 316 genes were up-regulated and 225 genes were down-regulated in receptive uterus compared to non-receptive uterus. Gene ontology and gene network analysis highlighted the activation of inflammatory response in the receptive uterus. By analyzing the promoter sequences of differentially expressed genes, we identified 12 causal transcription factors. Through connectivity map (CMap) analysis, we revealed several compounds with potential anti-receptivity activity. Finally, we performed a cross-species comparison against human uterine receptivity from a published dataset. Our study provides a valuable resource for understanding the molecular mechanism underlying uterine receptivity in mice.

## Introduction

Embryo implantation into the uterus is a crucial process for human pregnancy (Wang and Dey, 2006). Human embryo implantation is a relatively low-efficiency process. It has been demonstrated that the maximum chance of pregnancy occurring in a menstrual cycle is approximately 30%, largely due to implantation failure (Wilcox et al., 1993; Zinaman et al., 1996). Successful implantation requires both an implantation competent blastocyst and a receptive endometrium. In fact, although embryo defect is responsible for two thirds of implantation failures, inadequate uterine receptivity has been estimated to contribute to the other one third (Macklon et al., 2006). Therefore, it is imperative to understand the molecular mechanism underlying uterine receptivity.

Due to ethical restrictions and experimental difficulties, studies on human uterine receptivity are limited to descriptive ones which focus on gene expression levels. In addition to conventional gene-by-gene methods, in recent years, various high-throughput profiling approaches make it possible for simultaneously studying the expression level of thousands of genes. Global gene expression changes associated with uterine receptivity have been determined by 10 independent groups (Carson et al., 2002; Kao et al., 2002; Borthwick et al., 2003; Riesewijk et al., 2003; Mirkin et al., 2005; Talbi et al., 2006; Diaz-Gimeno et al., 2011; Altmae et al., 2012, 2017; Hu et al., 2014). Notably, serval *in vitro* systems have been established to study the molecular mechanism of human uterine receptivity (Rahnama et al., 2009; Huang et al., 2017). However, a cell layer growing in a dish may not resemble the *in vivo* condition. Moreover, the uterus is comprised of many cell types. Cultured cells are lack of interacting microenvironment. *In vivo* analysis of uterine receptivity heavily relies on the mouse. As revealed by gene knockout mice, a number of genes have been implicated in mouse uterine receptivity and embryo implantation. These include Esr1 (estrogen receptor 1) (Curtis Hewitt et al., 2002), Lif (leukemia inhibitory factor) (Stewart et al., 1992), Hoxa10 (homeobox A10) (Bagot et al., 2001), Hoxa11 (homeobox A11) (Gendron et al., 1997), Msx1 (msh homeobox 1) (Daikoku et al., 2011), and Ihh (Indian hedgehog) (Lee et al., 2006). Although global gene expression changes at the implantation site compared to the inter-implantation site have been investigated repeatedly (Liu et al., 2011), studies with regard to mouse uterine receptivity are scarce. In one study, microarray was used to determine the global gene expression profile in uterine luminal epithelium enzymatically isolated before and post implantation (Xiao et al., 2014). In another study, uterine luminal epithelium enzymatically isolated from pseudo-pregnant mouse was examined by microarray and gene expression levels were determined from days 3 to 5 (Campbell et al., 2006).

In the present study, using the RNA-seq approach, we analyzed global gene expression changes in receptive uterus on day 4 of pregnancy compared to non-receptive uterus on day 3 of pregnancy in mice. RNA-seq is highly accurate in quantifying genome-wide gene expression levels. Compared to the microarray, the main advantages of RNA-seq are: the ability to detect un-annotated transcripts (Wang et al., 2009), discriminating very similar sequences (Mortazavi et al., 2008), and no upper limit for quantification (Garber et al., 2011). Our study may contribute to an increase in the knowledge on uterine receptivity.

## Materials and Methods

### Sample Collection

CD-1 mice were used for this study. Natural pregnancy was established by mating adult females with fertile males. The day of the observation of vaginal plug was recorded as day 1 of pregnancy. The whole uterus was obtained on day 3 (pre-receptive/non-receptive) and day 4 (receptive) of pregnancy. Success of pregnancy was confirmed by recovering embryos from the oviduct (on day 3) or the uterus (on day 4). All collected uterine samples were snap-frozen in liquid nitrogen and stored at -80°C until use. All animal procedures in this study were approved by the Institutional Animal Care and Use Committee of South China Agricultural University.

### RNA-seq

The TRIzol reagent (Invitrogen) was used to extract total RNA. The purity and integrity of total RNA was assessed by using the ND-1000 Nanodrop and the Agilent 2200 TapeStation with the following quality control parameters: A260/A280 ratio > 1.8, A260/A230 ratio > 2.0 and RNA integrity number (Schroeder et al., 2006) value > 7.0. RNA-seq libraries were generated by using the TruSeq RNA sample preparation kit (Illumina). High-throughput sequencing was performed using the Illumina HiSeq 2500 system. After sequencing, raw data were processed by a computational pipeline as described previously (Huang et al., 2018). Raw data were first aligned to mouse genome (UCSC mm9) using TopHat v2.0.4 with default options (Trapnell et al., 2009) and then assembled using Cufflinks v2.2.1 (Trapnell et al., 2010). Differentially expressed genes were chosen based on fold change >2 and *P* < 0.05.

### Validation by Quantitative RT-PCR

The TRIzol reagent (Invitrogen) was used to extract total RNA. Potential genomic DNA contamination was eliminate by DNase I treatment (Invitrogen). The synthesis of cDNA was conducted using the PrimeScript reverse transcriptase reagent kit (TaKaRa). Quantitative RT-PCR was performed using THUNDERBIRD SYBR qPCR Mix (Toyobo) on the Applied Biosystems 7500 (Life Technologies). The Rpl7 gene served as a reference gene for normalization. Primer sequences used in this study were listed in Supplementary Table S1.

### Gene Ontology (GO) and Pathway Analysis

Gene Ontology and pathway analysis was performed by using the DAVID online tools (Huang et al., 2007). The significance cutoff for FDR was set at 0.05. The word cloud for significantly enriched GO and pathway terms was created by using the R package wordcloud.

### Gene Network Construction

The gene network was constructed by using the STRING v10.0 database (Szklarczyk et al., 2015). The minimum combined score of the hub gene network was set to 0.4 by default. The Cytoscape software (Shannon et al., 2003) was applied for view and analysis of the gene network. The Cytoscape plugin Network Analyzer (Assenov et al., 2008) was employed to calculate the degree distribution. The mean plus two standard deviations was chosen as the degree threshold value for hub genes.

### Analysis of Transcription Factor Binding Sites (TFBS)

The putative promoter sequences, which are defined as 1 kb upstream of transcription start site, were retrieved from the UCSC Genome Browser^1^. Position-weigh matrices (PWM) in the TRANSFAC database (Wingender et al., 1996) were searched by using the TESS software v6.0 (Schug, 2008). The relative score cutoff was 0.9. A hypergeometric test was conducted using in-house PERL scripts. A *P* < 0.01 was considered as an enriched transcription factor.

### Connectivity Map (CMap) Query

The up- and down-regulated genes were submitted simultaneously for CMap analysis^2^. The gene set enrichment analysis algorithm (Lamb et al., 2006) was used to calculate enrichment score for each compound.

## Results

### Identification of Gene Expression Changes Associated With Uterine Receptivity in Mice

**FIGURE 1:**
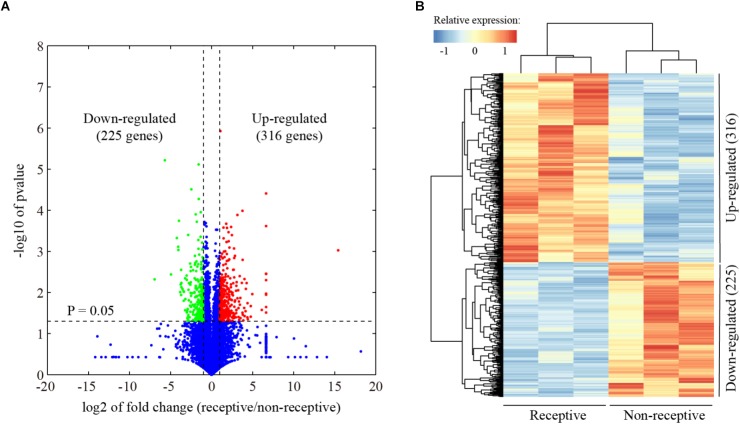
Identification of differentially expressed genes associated with endometrial receptivity. **(A)** Volcano plot for the comparison between the receptive endometrium (day 4 of pregnancy) and pre-receptive endometrium (day 3 of pregnancy) in mice. The cutoff values fold change >2 and FDR < 0.01 were utilized to identify differentially expressed genes. Non-changed genes were shown in blue color. Red color is indicative of up-regulated genes and green is indicative of down-regulated genes. **(B)** Heatmap plot of differentially expressed genes. The Pearson correlation distance metric and the average linkage clustering algorithm were used.

In order to capture global gene expression changes associated with uterine receptivity in mice, RNA-seq data were generated from the pre-receptive/non-receptive uterus on day 3 and receptive uterus on day 4 of pregnancy, with three biological replicates, respectively. Using a fold change cutoff of 2 and a *P*-value cutoff of 0.05, we identified a total of 541 differentially expressed genes (Figure 1A and Supplementary Table S2). Unsupervised hierarchical clustering analysis revealed that 316 genes were up-regulated and 225 genes were down-regulated in the receptive uterus compared to the non-receptive uterus (Figure 1B).

**FIGURE 2:**
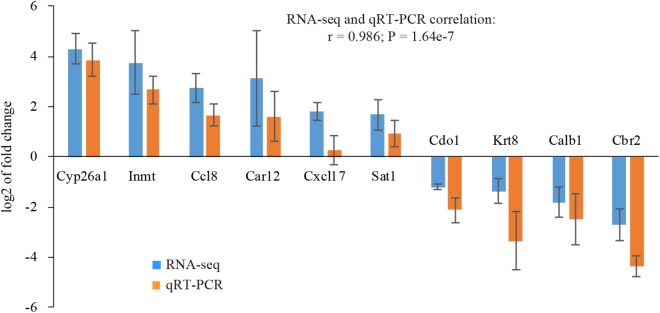
Validation of selected genes using qRT-PCR. Fold changes determined by RNA-seq and qRT-PCR were presented as the mean ± SEM. Statistical significance was reached at *P* < 0.05 for all genes except Cxcl17. *n* = 3.

In order to validate our RNA-seq data, we randomly selected 10 genes with various fold changes and subjected to quantitative RT-PCR (qRT-PCR) analysis. The validation was performed using an independent set of uterine samples. It turned out the expression pattern determined by qRT-PCR was accordant with our RNA-seq data (*r* = 0.986, *P* = 1.64e-7). All genes were confirmed to be significantly expressed (*P* < 0.05), except Cxcl17 (Figure 2), indicative of high quality of our RNA-seq data.

### Functional Clustering by Gene Ontology (GO) and Pathway Analysis

**FIGURE 3:**
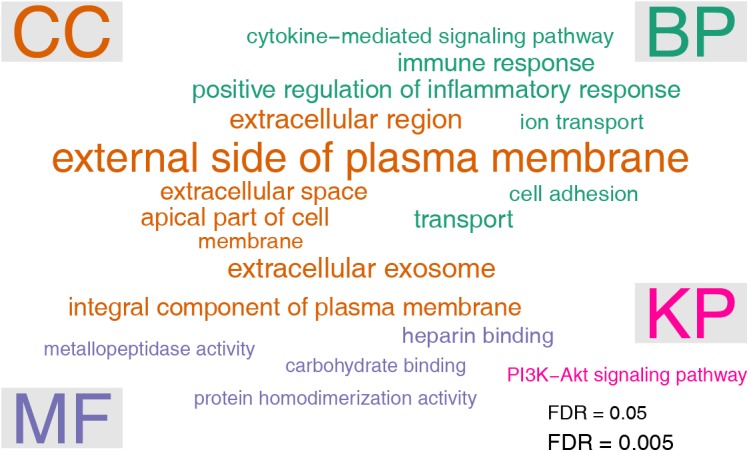
Gene ontology (GO) and pathway analysis of differentially expressed genes. The enrichment test was performed by using the DAVID tool. The significance cutoff for FDR was set at 0.05. The font sizes in the word cloud were proportional to –log10 of FDR. GO terms were arranged in three categories: biological process (BP), cellular component (CC), and molecular function (MF), respectively. Pathway analysis was based on KEGG pathway (KP) annotations.

Gene ontology analysis was performed by using the DAVID online tools. Enriched GO terms were grouped in the three categories: biological process (BP), cellular component (CC), and molecular function (MF), respectively. In the BP category, six terms were significantly enriched, including positive regulation of inflammatory response (FDR = 0.000532), transport (FDR = 0.00136), immune response (FDR = 0.00141), cell adhesion (FDR = 0.00592), ion transport (FDR = 0.00977), cytokine-mediated signaling pathway (FDR = 0.0235). The seven enriched GO terms under the CC category were membrane (FDR = 4.98e-9), external side of plasma membrane (FDR = 0.0000915), extracellular exosome (FDR = 0.000157), extracellular region (FDR = 0.00122), extracellular space (FDR = 0.00133), integral component of plasma membrane (FDR = 0.00216), and apical part of cell (FDR = 0.0351). With respect to the MF category, four terms were significantly enriched, including protein homodimerization activity (FDR = 0.0302), heparin binding (FDR = 0.0313), carbohydrate binding (FDR = 0.0432), and metallopeptidase activity (FDR = 0.0487). We also performed pathway analysis by using the DAVID online tools. It turned out that only one pathway, namely PI3K-Akt signaling pathway (FDR = 0.0387), was significantly enriched (Figure 3).

### Prioritization of Differentially Expressed Genes in Gene–Gene Network

**FIGURE 4:**
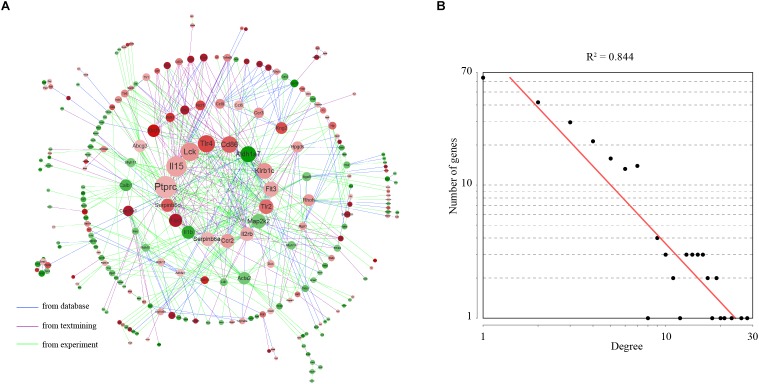
Gene network underlying differentially expressed genes. **(A)** The structure of the gene–gene interaction network. Up-regulated genes were colored in red and down-regulated genes were colored in green. The 16 hub genes were showed in the center of the network. Hub genes were defined as genes with degree values exceeding the mean plus two standard deviations. **(B)** Degree distribution of the network.

The STRING database was employed to analyze the gene network for differentially expressed genes. We constructed a gene network containing 289 nodes and 682 edges (interaction score > 0.4) (Figure 4A). Topological analysis indicated that this gene network was a scale-free network (Barabasi and Oltvai, 2004; Figure 4B). In a scale-free network, only a few nodes, known as hub genes, have a very high degree of connection, whereas the majority of nodes exhibits a low degree of connection. Using a defined cut-off value, we identified a total of 18 hub genes. Considering their key positions in the gene network, the hub genes are expected to be likely more important than the other genes.

### Inferring Regulatory Mechanisms Underlying Differentially Expressed Genes

**FIGURE 5:**
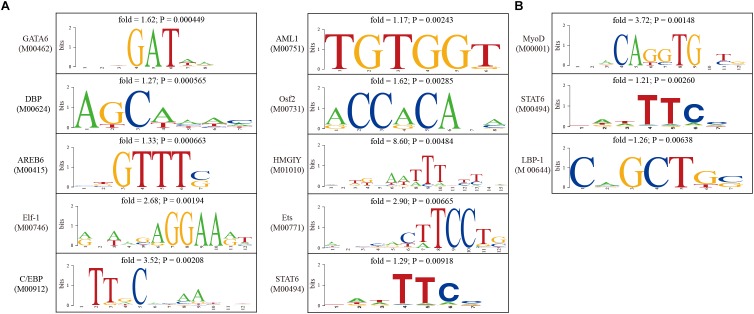
Analysis of transcription factor binding sites in the promoter of differentially expressed genes. **(A)** The sequence logos for transcription factors whose binding sites were significantly enriched in the promoter of up-regulated genes. **(B)** The sequence logos for transcription factors whose binding sites were significantly enriched in the promoter of down-regulated genes.

Gene expression are largely controlled by transcription factors. In order to indentify causal transcription factors for differentially expressed genes, transcription factor binding sites were predicted using the TESS software. Enrichment of transcription factor binding sites were tested separately for up-regulated genes and down-regulated genes. We found that the binding sites of GATA6, DBP, AREB6, Elf-1, C/EBP, AML1, Osf2, HMGIY, Ets, and STAT6 were significantly over-represented among up-regulated genes (Figure 5A), whereas the binding sites of MyoD, STAT6, and LBP-1 were significantly over-represented among up-regulated genes (Figure 5B). These findings provided insights into the regulatory mechanisms underlying uterine receptivity in mice.

### Searching for Anti-receptivity Chemical Drugs via Connectivity Map (CMap)

**FIGURE 6:**
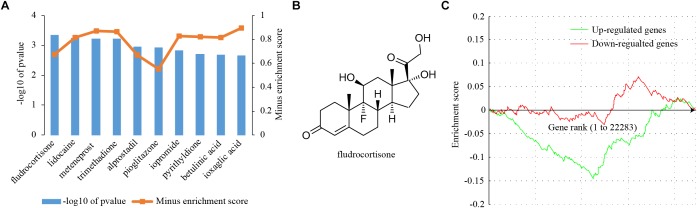
Connectivity map (CMap) analysis. **(A)** The enrichment scores of the top 10 chemical drugs from CMap analysis. Differentially expressed genes were queried into CMap and chemical drugs showing a negative enrichment score were considered. **(B)** The molecular structure of the top-ranked chemical drug, fludrocortisone. **(C)** A graphical view of the enrichment score for fludrocortisone. The enrichment score is determined by computing a Kolmogorov–Smirnov (KS) statistic separately for the up- and down-regulated genes.

Chemical drugs that are able to reverse the expression of receptivity-related genes may exert anti-receptivity effects. To this end, a CMap analysis was performed to search for drugs that had a negative gene expression pattern for uterine receptivity. The Kolmogorov–Smirnov (KS) statistic was computed for both up- and down-regulated genes, respectively. If the KS statistic for up-regulated genes and down-regulated genes were in the same direction, the connectivity score was set to zero; otherwise the connectivity score was set to the KS statistic for up-regulated genes minus the KS statistic for down-regulated genes. Connectivity scores were used to compute a permuted *P*-value for each drug. The top 10 most promising repositioned chemical drugs according to permuted *P*-values were shown in Figure 6A. Fludrocortisone was the most promising drug (Figure 6B). The best connectivity scores for fludrocortisone in up-regulated genes and down-regulated genes were -0.15 and 0.072, respectively (Figure 6C), resulting a combined connectivity score of -0.795. We found that a total of 189 genes could be potentially reversed by fludrocortisone, of which 61 genes whose expression was up-regulated in receptive uterus could be repressed and 127 genes whose expression was down-regulated in receptive uterus could be induced (Supplementary Table S3). Fludrocortisone is a synthetic corticosteroid with mineralocorticoid and glucocorticoid activity. The idea that fludrocortisone can be repurposed into an anti-receptivity drug deserves further investigation.

### Comparison of Uterine Receptivity Between Mice and Humans

**FIGURE 7:**
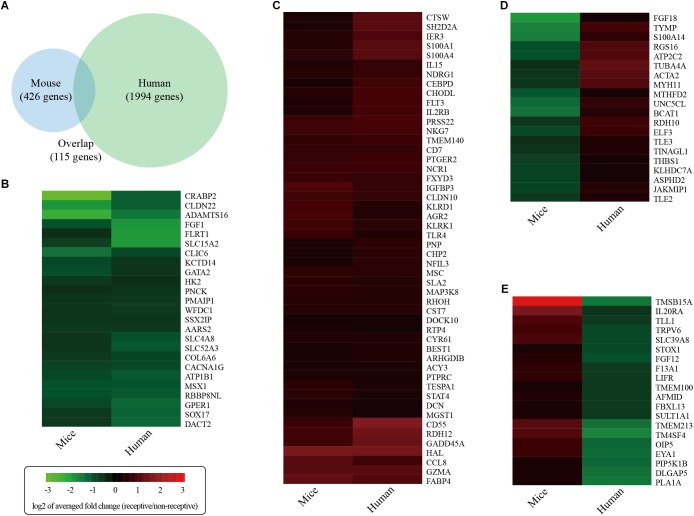
Global comparison of the gene expression changes associated with endometrial receptivity in mice against humans. **(A)** Venn diagram showing the overlap of differentially expressed genes between mice and humans. **(B)** Consistently down-regulated genes. **(C)** Consistently up-regulated genes. **(D)** Inconsistently expressed genes that were down-regulated in mice but up-regulated in humans. **(E)** Inconsistently expressed genes that were up-regulated in mice but down-regulated in humans. Heatmaps were draw according to log2 of averaged fold change values.

To identify differentially expressed genes associated with uterine receptivity in humans, we re-analyzed a published RNA-seq dataset on receptive endometrium (LH+8) and pre-receptive endometrium (LH+2) from the same 20 fertile women (GSE98386) (Altmae et al., 2017). We identified a total of 2109 differentially expressed genes. Comparative analysis revealed that 115 genes were shared by both the human and the mouse (Figure 7A). Among these 115 genes, 25 genes were consistently down-regulated (Figure 7B) and 50 genes were consistently up-regulated (Figure 7C) in mice and humans. However, 20 genes were down-regulated in mice but up-regulated in humans (Figure 7D). In addition, there were 20 genes up-regulated in mice but up-regulated in humans (Figure 7E). These data highlight the difference in uterine receptivity between mice and humans.

## Discussion

The uterus is receptive during a restricted “window of implantation” (Yoshinaga, 1988). In the mouse, the receptive period is limited to day 4 of pregnancy. The uterus is not receptive to embryo implantation on days 1 to 3. The uterus immediately enters a refractory phase on day 5 (Zhang et al., 2013). In this study, we investigated the gene expression profile in receptive uterus on day 4 compared with non-receptive uterus on day 3 of pregnancy using RNA-seq. A total of 541 genes, including 316 up-regulated and 225 down-regulated genes, were identified to be differentially expressed in receptive uterus compared with non-receptive uterus. Quantitative RT-PCR analysis demonstrated that the expression pattern of serval selected genes was consistent with RNA-seq data, indicative of high quality of our RNA-seq data.

Furthermore, a systematic and comprehensive literature search was performed for top 10 up-regulated genes according to fold change value in the PubMed database. Among these 10 genes, the expression pattern of two genes have been reported in the mouse uterus during the peri-implantation period. In our RNA-seq data, the expression of Atp6v0d2 was up-regulated by 69.3 folds in the receptive uterus compared to non-receptive uterus, which was consistent with previous studies showing that Atp6v0d2 was highly expressed before implantation initiation (Xiao et al., 2014, 2017). Previous studies showed that the expression of Cyp26a1 mRNA was strongly induced from day 4 of pregnancy in mice (Vermot et al., 2000; Ma et al., 2012). The expression of Cyp26a1 was up-regulated by 20.7 folds in the receptive uterus compared to non-receptive uterus in our RNA-seq data. Additionally, we found two well-known down-regulated genes, Gata2 (Rubel et al., 2012) and Msx1 (Nallasamy et al., 2012), in our RNA-seq data. These findings may provide validity of our RNA-seq data.

One clear limitation of this study is that the whole uterus is used for RNA-seq analysis. The uterine wall consists of three layers, endometrium, myometrium, and perimetrium. Although the perimetrium is very thin, the myometrium is thick and thus may dilution gene expression changes in the endometrium. In the pre-experiment phase of this study, we isolated the endometrium from the whole uterus by squeezing with a bent syringe needle on a glass slide. Endometrial samples were subjected to RNA-seq. We identified a total of 93 differentially expressed genes (Supplementary Table S4). However, further validation showed large variation between samples, likely due to the incomplete removal of myometrium or the unwanted loss of endometrial tissue during the squeezing process. In order to increase the reproducibility of the data, we decided to use the whole uterus in this study. The myometrium is generally considered as a quiescent tissue before parturition and genes differentially expressed in myometrium may be scarce. Notably, there are many cell types in endometrium, luminal and glandular epithelial cells, stromal cells, endothelial cells, and various immune cells. Enzymatically isolated uterine luminal epithelium was used for microarray analysis (Campbell et al., 2006; Chen et al., 2006; Xiao et al., 2014); the disadvantage of this approach is that gene expression levels are likely altered in enzymatically isolated cells compared to normal physical conditions. It is undoubtedly that application of laser-capture microdissection-based (Yoon et al., 2004) or single-cell based (Krjutskov et al., 2016) RNA-seq will be a better choice for this study. However, the compromised sensitivity is the limiting factor for these two approaches at the moment. Thus, this bulk tissue RNA-seq study may provide an irreplaceable resource for in-depth understanding of uterine receptivity.

Uterine receptivity is mainly under the control of ovarian steroids. However, we demonstrated previously that the preimplantation floating embryo significantly affected the expression of 223 genes (Liu, 2018). Many of these genes were involved in immune response. Interestingly, we found that only three embryo-induced genes, Aqp5 (aquaporin 5), Mycn (v-myc avian myelocytomatosis viral related oncogene), and F5 (coagulation factor V), were associated with uterine receptivity based on RNA-seq data of this study. Our data suggest that the preimplantation floating embryo may not have a significant impact on uterine receptivity.

Gene ontology (GO) and pathway analysis was performed to explore the functions of differentially expressed genes. Interestingly, we found inflammatory response was the most enriched term under the BP category of GO. The inflammatory marker Ptgs2 (prostaglandin-endoperoxide synthase 2) is significantly elevated in receptive endometrium compared with prereceptive endometrium in humans and monkeys (Marions and Danielsson, 1999; Sun et al., 2004). In mice, the pro-inflammatory Lif (leukemia inhibitory factor) transiently increases in mouse uterus before implantation (Stewart et al., 1992). These data indicate that the endometrium before implantation is in an inflammatory state. L-selectin, which plays a key role in leukocyte capture from the bloodstream, is expressed by trophoblast cells of the blastocyst (Genbacev et al., 2003). The embryo implantation process is likely a mimicry of the leukocyte-endothelium interaction: by acting like a leukocyte, the embryo sticks and migrates into the “inflamed” endometrium (Liu, 2018). Hence, inflammation is a mechanism of uterine receptivity. Additionally, we found that PI3K-Akt signaling pathway was the only enriched pathway. Intrauterine injection of the PI3K/Akt inhibitor LY294002 on day 2 of pregnancy impaired embryo implantation in mice (Liu et al., 2014). Mechanically, PI3K/Akt inhibition resulted in reciprocal activation of Sgk1 (glucocorticoid regulated kinase 1). Down-regulation of Sgk1 in the receptive uterus is required for embryo implantation (Salker et al., 2016). Therefore, the activation of PI3K-Akt signaling pathway may represent a critical event in the establishment of uterine receptivity. Network analysis was performed to identify 18 hub genes, including three down-regulated genes Aldh1a7 (aldehyde dehydrogenase family 1 subfamily A7), Il1b (interleukin 1 beta), Map2k2 (mitogen-activated protein kinase kinase 2), and 15 up-regulated genes Ptprc (protein tyrosine phosphatase receptor type C), Il15 (interleukin 15), Lck (lymphocyte protein tyrosine kinase), Tlr4 (toll-like receptor 4), Cd86 (CD86 antigen), Flt3 (FMS-like tyrosine kinase 3), Tlr2 (toll-like receptor 2), Il2rb (interleukin 2 receptor, beta chain), Ccr2 (chemokine C-C motif receptor 2), Serpinb6a (serine/cysteine peptidase inhibitor clade B member 6A), Fasl (Fas ligand), Serpinb6c (serine/cysteine peptidase inhibitor clade B member 6C). The hub genes are expected to be more important than other genes in the network. According to gene ontology, all these hub genes expect Aldh1a7 and Map2k2, are involved in inflammatory response process. Thus, the network analysis highlighted the role of inflammatory response in uterine receptivity.

Furthermore, causal transcription factors which might drive the expression of differentially expressed genes were predicted by enrichment test. We found that the binding sites of GATA6, DBP, AREB6, Elf-1, C/EBP, AML1, Osf2, HMGIY, Ets, and STAT6 were significantly over-represented among up-enriched genes, whereas MyoD, STAT6 and LBP-1 binding sites were significantly over-represented among down-regulated genes. GATA6 (GATA-binding factor 6) is a member of the GATA family of zinc finger transcription factors that are characterized by their DNA binding domain (Maeda et al., 2005). GATA6 is expressed in the adult mouse uterus (Freyer et al., 2015). DBP (albumin D-element-binding protein) is a circadian transcriptional factor. The circadian rhythm is likely required for embryo implantation (Pilorz and Steinlechner, 2008; Muter et al., 2015). AREB6 is officially known as ZEB1 (zinc finger E-box binding homeobox 1). ZEB1 is expressed in myometrial and stromal parts of mouse uterus on day 5 of pregnancy (Spoelstra et al., 2006). Elf-1 is known as E74-like ETS transcription factor 1. The ETS (E26 transformation specific) is a family of transcription factors that are capable of regulating transcription by binding to ETS-binding sites [5′-GGA(A/T)-3′] in the promoter of target genes. Several members of ETS may participate in embryo implantation (Koo et al., 2005; Tabibzadeh, 2011). C/EBP (CCAAT-enhancer-binding protein) is a family of transcription factors composed of 6 members, named from C/EBPα to C/EBPζ. C/EBPβ-null female mice are infertile. It was demonstrated that estrogen-induced epithelial cell proliferation was markedly compromised in the absence of C/EBPβ (Mantena et al., 2006). AML1 (officially known as RUNX1) and Osf2 (officially known as RUNX2) are runt-related transcription factors. Both of them are dynamically expressed in mouse uterus during embryo implantation (Bai et al., 2015; Guo et al., 2016). HMGIY is officially known as HMGA1 (high mobility group AT-hook 1). HMGA1 functions as an oncogene in uterine tumorigenesis by activating PTGS2 expression (Tesfaye et al., 2007). STAT6 (signal transducer and activator of transcription 6) is a member of the STAT family of transcription factors. Notably, STAT6 binding sites were commonly enriched for both down-regulated and up-regulated genes. Conditional ablation of STAT3, a paralog of STAT6, in mouse uterus impairs uterine receptivity and decidualization (Lee et al., 2013; Pawar et al., 2013; Sun et al., 2013). MyoD (myogenic differentiation 1) is a transcription factor binding to a DNA motif known as the E-box (enhancer box). LBP-1 (upstream binding protein 1) is a member of the NTF (neurogenic element-binding) family of transcription factors. Currently, the role of MyoD and LBP-1 in regulating uterine gene expression is unknown. These causal transcription factors may deserve further investigation.

By CMap analysis, we identified compounds with a reverse gene expression profile to differentially expressed genes. The top 10 most promising compounds were: fludrocortisone, lidocaine, meteneprost, trimethadione, alprostadil, pioglitazone, iopromide, pyrithyldione, betulinic acid, and ioxaglic acid. Fludrocortisone is a synthetic corticosteroid with mineralocorticoid and glucocorticoid activity. In mice, estrogen is a critical determinant for uterine receptivity (Ma et al., 2003). The antagonism of glucocorticoids and estrogens in the mouse uterus has been reported (Rabin et al., 1990; Rhen et al., 2003). Therefore, the anti-receptivity effect of fludrocortisone may attribute to its glucocorticoid activity. Lidocaine is a medication used to numb tissue. Lidocaine is a blocker of the fast voltage-gated Na^+^ channels in the neuronal cell membrane. Alprostadil is a naturally occurring prostaglandin E1 (PGE1) and meteneprost is a potent analog of prostaglandin E2 (PGE2). Paradoxically, PGE2 might promote implantation by improving endometrial receptivity (Huang et al., 2017). Trimethadione is a dione-type anticonvulsant, which reduces T-type calcium currents in thalamic neurons. Pioglitazone is a drug with hypoglycemic action to treat diabetes. Pioglitazone is a selective agonist for nuclear receptor peroxisome proliferator-activated receptor gamma (PPARγ) and to a lesser extent PPARα (Gillies and Dunn, 2000). PPARs may play important roles in mouse uterus during early pregnancy (Nishimura et al., 2011). Pyrithyldione is a psychoactive drug. Betulinic acid is a naturally occurring pentacyclic triterpenoid as an anticancer agent by inhibition of topoisomerase (Chowdhury et al., 2002). Iopromide and ioxaglic acid are iodine containing molecules used as a low-osmolality contrast medium. According to literature, there is high possibility that fludrocortisone is an anti-receptivity drug. The remaining drugs, which are seemingly unrelated to uterine receptivity so far, may provide insights into the development of novel anti-receptivity drugs.

The idea that fludrocortisone can be repurposed into an anti-receptivity drug deserves further investigation. Further *in silico* analysis revealed that fludrocortisone could potentially reverse 189 genes: 61 genes are repressed and 127 genes are induced. Of interest, msh homeobox 1 (Msx1), which is down-regulated in the receptive uterus according to our RNA-seq data, could be induced by fludrocortisone. The expression level of Msx1 is very low in the uterus of non-pregnant mice. It increases dramatically on day 3 of pregnancy, and rapidly decreases before implantation (Nallasamy et al., 2012). Similarly, in the human endometrium, MSX1 expression appears to be down-regulated before the window of implantation (Kao et al., 2002; Riesewijk et al., 2003; Mirkin et al., 2005; Burmenskaya et al., 2017). Conditional ablation of Msx1 impaired embryo implantation in mice (Daikoku et al., 2011; Nallasamy et al., 2012). Msx1 is highly expressed in the uterus of experimentally induced delayed implantation mouse model and becomes undetectable upon implantation activation (Cha et al., 2013). Strikingly, mice with conditional uterine ablation of Msx1 fail to undergo delayed implantation and implantation-like response can be found at the site of the blastocyst in the delayed implantation model (Cha et al., 2013, 2015). This finding indicates that the down-regulation of Msx1 prior to implantation may be a prerequisite for the establishment of uterine receptivity. Therefore, reversing Msx1 expression is likely a mechanism for the anti-receptivity activity of fludrocortisone.

In humans, the uterine receptivity period occurs between days 20 and 24 (from LH+6 to LH+10) of a regular 28-day menstrual cycle. Global gene or protein expression changes associated with uterine receptivity have been determined by independent groups (Carson et al., 2002; Kao et al., 2002; Borthwick et al., 2003; Riesewijk et al., 2003; Mirkin et al., 2005; Talbi et al., 2006; Diaz-Gimeno et al., 2011; Altmae et al., 2012, 2017; Hu et al., 2014). However, little consistency is observed in these studies (Horcajadas et al., 2007). In this study, we re-analyzed a published RNA-seq dataset on receptive endometrium (LH+8) and pre-receptive endometrium (LH+2) from the same 20 fertile women (GSE98386) (Altmae et al., 2017). This dataset was chosen, because (a) the sample size was larger than the others, (b) the same patient was recruited to collect LH+8 and LH+2 samples, and (c) RNA-seq was employed which is more accurate than the microarray approach. We identified a total of 2109 differentially expressed genes. Comparative analysis revealed that 115 genes were shared by both humans and mice. Among these 115 genes, 75 genes were consistently expressed, whereas 40 genes inconsistently expressed between mice and humans. These data suggest that uterine receptivity is not congruent in some aspects between mice and humans. Nevertheless, we would like to note that the human dataset was obtained using endometrial biopsy without the myometrium layer, whereas our mouse data were collected form the whole uterus including myometrium and perimetrium. Therefore, it is possible that this comparative analysis might exaggerate the differences of uterine receptivity between humans and mice.

In conclusion, in the present study, using RNA-seq, we investigated the gene expression profile in receptive uterus on day 4 compared with non-receptive uterus on day 3 of pregnancy. Our study provides a valuable resource for understanding of the molecular mechanisms underlying uterine receptivity.

## Author Contributions

J-LL conceived and designed the experiments. J-PH and J-LL performed the experiments and analyzed the data. MZ, W-QZ, M-YH, CZ, and H-ZC contributed analysis tools. J-PH and J-LL wrote the manuscript.

## Conflict of Interest Statement

The authors declare that the research was conducted in the absence of any commercial or financial relationships that could be construed as a potential conflict of interest.

## Footnotes

1 http://genome.ucsc.edu/

2 http://www.broadinstitute.org/cmap/
